# Point-of-care Ultrasound Diagnosed Intraocular Breast Metastasis

**DOI:** 10.5811/cpcem.1686

**Published:** 2024-05-14

**Authors:** Hamzah M. Yusuf, Timothy Batchelor, Nicholas Ashenburg

**Affiliations:** Stanford University, Department of Emergency Medicine, Palo Alto, California

**Keywords:** *POCUS*, *ocular ultrasound*, *emergency medicine*, *breast cancer*, *metastasis*

## Abstract

**Case Presentation:**

A 60-year-old female presented to the emergency department with unilateral eye pain and vision loss. Point-of-care ultrasound (POCUS) was performed, which demonstrated ocular metastatic lesions of breast cancer.

**Discussion:**

Ocular metastasis is rare, clinically challenging, and may present with a wide range of ophthalmic symptoms. However, POCUS may safely and rapidly identify metastatic lesions to direct further care.

Population Health Research Capsule What do we already know about this clinical entity?

*Ocular metastasis represents a rare, clinically challenging manifestation of advanced cancer.*

What is the major impact of the image(s)?

*We describe an example of breast cancer metastasis on point-of-care ultrasound (POCUS), its clinical manifestations, and potential treatments.*

How might this improve emergency medicine practice?

*Emergency physicians may be able to diagnose ocular malignancy using POCUS, a fast and easy-to-use bedside tool.*

## CASE PRESENTATION

A 60-year-old female with a history of recently diagnosed breast cancer with liver, lung, and bone metastases pending biopsy results presented to the emergency department with one day of progressive left eye pain, blurry vision, and central scotoma. She denied myodesopsia, photopsia, or recent eye trauma. Fluorescein staining was unremarkable. Her visual acuity was 20/30 bilaterally. She had intraocular pressures of 19 millimeters of mercury (mm Hg) bilaterally (reference range 10–20 mm Hg), and equally round and reactive pupils. She had full extraocular movement. Her slit lamp exam was notable for mild, left eye conjunctival injection.

Using a linear ultrasound probe, we performed point-of-care ultrasound (POCUS) to obtain sagittal and transverse views evaluating the anterior chamber, posterior chamber, retina, lens location, and overall structure of the left eye. Two posterior masses were identified over the optic nerve (Image). Ophthalmology was consulted and, on dilated fundus exam, identified two, pale yellow choroidal lesions in close proximity to the optic nerve and optic nerve tilting, with strong concern for leptomeningeal involvement. The patient was then admitted to the hospital for enhanced imaging and facilitation of care.

**Image.**
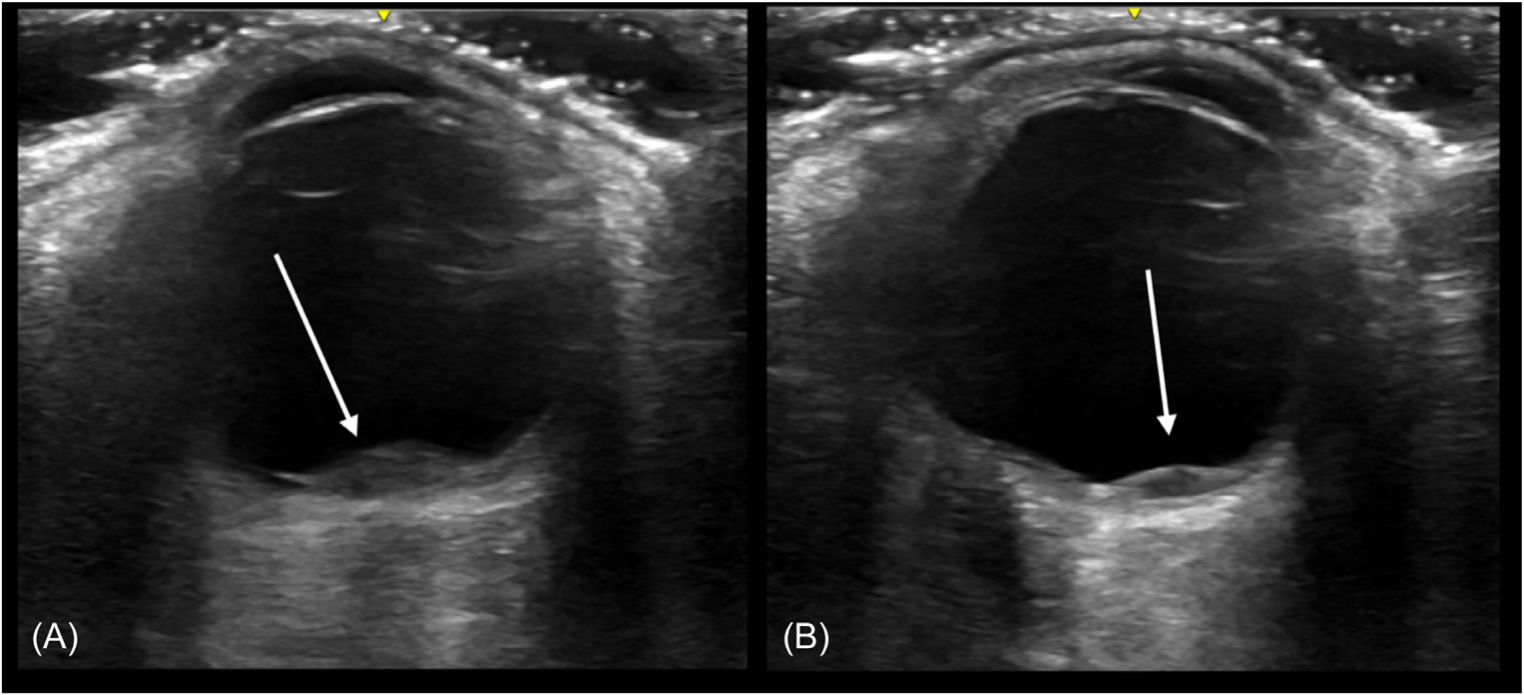
Point-of-care ultrasound using liner 8-megahertz transducer in transverse view demonstrating, (A) hypoechoic lenticular-shaped optic metastasis (3.2 millimeters [mm] by 1.5 mm) over the optic nerve, and (B) a smaller lenticular-shaped metastasis (1.7 mm by 1.0 mm) with the patient in leftward gaze.

An inpatient magnetic resonance imaging study of the brain with and without contrast demonstrated hypointense nodularity in several areas within the globe, with several, scattered, small enhancing cerebral lesions. She was started on corticosteroids and offered whole brain vs stereotactic body radiation therapy, as well as external beam therapy for intraocular tumors. She was subsequently discharged for outpatient radiology-oncology care.

## DISCUSSION

Ocular metastasis represents a rare and clinically challenging manifestation of advanced cancer. Breast cancer remains a more prevalent source of ocular metastasis due to its high incidence, aggressive nature, and propensity for systemic dissemination. The ocular structures, including the choroid, retina, iris, and optic nerve, serve as potential sites for secondary tumor growth, leading to visual impairment and significant morbidity.^1^ A POCUS exam may show a hypoechoic dome or plateau-shaped lesion typically over the choroid with minimal internal vascularization.^2^

Clinically, ocular metastasis can manifest as a wide range of ophthalmic symptoms, including blurred vision, pain, photophobia, metamorphopsia, or it can be asymptomatic.^1^ ^,^ ^3^ Estimating the incidence of ophthalmic metastasis has proven challenging as asymptomatic manifestations may go unnoticed, but the prevalence of ocular metastasis in metastatic breast cancer has been estimated between 9.7–30%.^4^ Differential diagnoses may include primary ocular malignancies, hemangioma, sclerochoroidal calcification, granuloma, and infection, underscoring the importance of accurate diagnosis to guide appropriate management strategies.

Subclinical metastasis may not indicate targeted treatment. At initial diagnosis, patients may receive systemic adjuvant treatment to eliminate micrometastatic disease and prevent further metastasis.^5^ Systemic treatment may include endocrine treatment, cytotoxic chemotherapy, corticosteroids, or immunotherapy. Targeted treatment to ocular metastases includes external beam radiation, which can be useful in patients with foveal involvement but may result in cataracts or radiation retinopathy.

The authors attest that their institution requires neither Institutional Review Board approval, nor patient consent for publication of this case report. Documentation on file.
